# Constitutive, Muscle-Specific *Orai1* Knockout Results in the Incomplete Assembly of Ca^2+^ Entry Units and a Reduction in the Age-Dependent Formation of Tubular Aggregates

**DOI:** 10.3390/biomedicines12081651

**Published:** 2024-07-24

**Authors:** Alessia Di Fonso, Matteo Serano, Miao He, Jennifer Leigh, Giorgia Rastelli, Robert T. Dirksen, Feliciano Protasi, Laura Pietrangelo

**Affiliations:** 1 Center for Advanced Studies and Technology (CAST), University G. d’Annunzio of Chieti-Pescara, I-66100 Chieti, Italy; alessia.difonso@unich.it (A.D.F.); matteo.serano@unich.it (M.S.); giorgia.rastelli@unich.it (G.R.); feliciano.protasi@unich.it (F.P.); 2 Department of Medicine and Aging Sciences (DMSI), University G. d’Annunzio of Chieti-Pescara, I-66100 Chieti, Italy; 3 Department of Pharmacology and Physiology, University of Rochester School of Medicine and Dentistry, Rochester, NY 14642, USA; miao_he@urmc.rochester.edu (M.H.); jennifer_leigh@urmc.rochester.edu (J.L.); robert_dirksen@urmc.rochester.edu (R.T.D.); 4 Department of Neuroscience and Clinical Sciences (DNISC), University G. d’Annunzio of Chieti-Pescara, I-66100 Chieti, Italy

**Keywords:** sarcoplasmic reticulum, skeletal muscle, store-operated Ca^2+^ entry, tubular aggregate myopathy

## Abstract

Store-operated Ca^2+^ entry (SOCE) is a ubiquitous cellular mechanism that cells use to activate extracellular Ca^2+^ entry when intracellular Ca^2+^ stores are depleted. In skeletal muscle, SOCE occurs within Ca^2+^ entry units (CEUs), intracellular junctions between stacks of SR membranes containing STIM1 and transverse tubules (TTs) containing ORAI1. Gain-of-function mutations in *STIM1* and *ORAI1* are linked to tubular aggregate (TA) myopathy, a disease characterized by the atypical accumulation of tubes of SR origin. Moreover, SOCE and TAs are increased in the muscles of aged male mice. Here, we assessed the longitudinal effects (from 4–6 months to 10–14 months of age) of constitutive, muscle-specific *Orai1* knockout (cOrai1 KO) on skeletal muscle structure, function, and the assembly of TAs and CEUs. The results from these studies indicate that cOrai1 KO mice exhibit a shorter lifespan, reduced body weight, exercise intolerance, decreased muscle-specific force and rate of force production, and an increased number of structurally damaged mitochondria. In addition, electron microscopy analyses revealed (i) the absence of TAs with increasing age and (ii) an increased number of SR stacks without adjacent TTs (i.e., incomplete CEUs) in cOrai1 KO mice. The absence of TAs is consistent with TAs being formed as a result of excessive ORAI1-dependent Ca^2+^ entry.

## 1. Introduction

Ca^2+^ homeostasis in skeletal muscle fibers is finely regulated through a complex interplay of various mechanisms, including (i) Ca^2+^ release from the sarcoplasmic reticulum (SR) during excitation–contraction (EC) coupling [1]; (ii) Ca^2+^ re-uptake into the SR by sarco-endoplasmic reticulum Ca^2+^ ATP-ase (SERCA) pumps [2,3,4,5,6]; and (iii) store-operated Ca^2+^ entry (SOCE) that promotes the influx of external Ca^2+^ when SR stores are depleted [7]. During repetitive stimulation, loss of intracellular Ca^2+^ due to the extrusion of Ca^2+^ ions from the myoplasm by Na^+^-Ca^2+^ exchangers (NCXs) and plasma membrane Ca^2+^ ATP-ases (PMCAs) could lead to a decrease in the available Ca^2+^ to sequester into the SR, a factor that can contribute to muscle fatigue [1,8]. Consistent with the possibility that this could lead to a reduction in SR Ca^2+^ content and the activation of SOCE, Gissel and Clausen [9] used ^45^Ca^2+^ studies to show that significant Ca^2+^ influx occurs during sustained, repetitive muscle activation.

As in non-excitable cells, SOCE in skeletal muscle is coordinated by two main players [7,10,11]: STIM1 (stromal interaction molecule-1), a luminal endoplasmic reticulum (ER)/SR Ca^2+^ sensor [12,13,14], and ORAI1, a Ca^2+^ release-activated Ca^2+^ (CRAC) channel located in the surface membrane [15,16,17,18]. The activation of SOCE is initiated by Ca^2+^ store depletion, promoting oligomerization and cytoplasmic extension of STIM1 following the dissociation of Ca^2+^ ions from luminal STIM1 EF-hands, well-known Ca^2+^ binding motifs [19]. In turn, extended STIM1 oligomers activate ORAI1 in the plasma membrane, enabling Ca^2+^ entry [20,21].

Experimental evidence collected over the past decade has found that exercise promotes SOCE in the skeletal muscle fibers by promoting the formation of Ca^2+^ entry units (CEUs), dynamic junctions that assemble during exercise and disassemble during recovery [22,23]. CEUs are (i) small and few under sedentary control conditions [22]; (ii) constitutively assembled in fibers that lack calsequestrin 1 (CASQ1) protein expression [24], possibly due to the enhanced susceptibility for Ca^2+^ store depletion [25]; and (iii) absent in the muscles of aged sedentary mice [26], possibly due to the reduced activity-dependent enhancement of SOCE.

Under physiological conditions, ORAI1-dependent SOCE supports maintained force production during repetitive stimulation, promotes muscle fiber growth, and enhances the maintenance of oxidative, fatigue-resistant type I and type IIA fibers [22,23,24,27,28,29,30]. In addition, dysfunction in SOCE activity contributes to multiple pathophysiological conditions: (a) compromised muscle function in aging [26,31,32] and (b) myopathies, including muscular dystrophy (MD), malignant hyperthermia (MH), and tubular aggregate myopathy (TAM) [20,33].

TAM is a relatively rare disorder typically caused by gain-of-function mutations in both *STIM1* and *ORAI1* [34,35,36,37,38,39,40,41], and to a lesser degree, mutations in genes encoding for *CASQ1* and the type-1 ryanodine receptor (*RYR1*) [34,42,43]. Clinically, TAM presentation is highly variable (from asymptomatic creatine kinase elevation to significant limb weakness, exercise intolerance, cramps, and muscle pain) and may be slowly progressive. However, an increased presence of tubular aggregates (TAs) in the skeletal muscle fibers of affected individuals is a key pathological hallmark observed in all TAM cases. TAs originate from SR membranes [44], as they stain positive for numerous SR proteins, including SERCA, triadin, and CASQ1 [45,46,47]. Also, the accumulation of some sarcolemmal proteins, including the dihydropyridine receptor (DHPR) and ORAI1, has been reported [26,43], though external membranes (i.e., transverse tubules or TTs) are excluded from TAs [45]. TAs are also observed in fast-twitch fibers of the extensor digitorum longus (EDL) muscles in aged male animals [45,46,47]. In mice, TAs are observed under conditions of altered SOCE activity (e.g., aging and TAM), which could contribute to both muscle weakness and increased susceptibility to fatigue. Interestingly, long-term voluntary wheel running exercise significantly reduces the incidence of TAs in aged male mice [26]. However, the precise molecular and morphological mechanisms that underlie the formation and stability of TAs are not fully understood.

Carrell et al. [27] described how slow-twitch soleus muscles from adult skeletal muscle-specific *Orai1* knockout (KO) mice (cOrai1 KO) exhibit a significant reduction in myofiber cross-sectional area (CSA) and muscle mass, as well as the replacement of some fatigue-resistant type I fibers with hybrid fibers expressing both type I and type IIA myosins [27]. These findings are consistent with ORAI1 being important for the muscle fibers in the soleus to properly undergo the postnatal transition from fast to slow myosin expression, such that the transitioning fibers become suspended in a hybrid state [48]. In contrast, fast-twitch EDL muscles from these mice exhibited a smaller reduction in muscle mass and CSA without significant fiber type changes. Additionally, ex vivo peak specific force production was reduced in excised muscles and in vivo exercise endurance was compromised in cOrai1 KO mice [27]. 

In the present study, we assessed the longitudinal effects of skeletal muscle-specific *Orai1* KO by comparing the muscle structure and function between 4–6-month-old and 10–14-month-old control and cOrai1 KO mice. The results from these studies indicate that cOrai1 KO mice exhibit a shorter lifespan, reduced body weight, exercise intolerance, decreased muscle-specific force and rate of force production, and an increased number of structurally damaged mitochondria. Unexpectedly, *Orai1* gene ablation in the skeletal muscle prevents the formation of TAs with increasing age and increases the presence of SR stacks without the association of TTs, as occurs with CEUs that promote SOCE [22].

## 2. Materials and Methods

### 2.1. Animals

cOrai1 KO mice were generated as previously described [27]. *Orai1*-floxed mice lacking Cre-recombinase were used as the controls. All the mice were from a congenic C57bl/6N background. The animals were housed in microisolator cages at 20 °C with a 12 h light/dark cycle and provided free access to standard chow. The in vivo procedures and experiments were conducted according to the National Committee for the Protection of Animals used for Scientific Purposes (D. lgs n.26/2014) and approved by the Italian Ministry of Health (AN 313/2019-PR) or the University Committee on Animal Resources at the University of Rochester (UCAR2006-114E). Ex vivo experiments were performed on EDL muscles dissected from euthanized control and cOrai1 KO mice. The animals were euthanized by cervical dislocation, as approved by the Italian D. lgs n.26/2014 and the University Committee on Animal Resources at the University of Rochester (UCAR2006-114E).

### 2.2. Age-Dependent Survival Analysis of Animals Housed under Standard Conditions

The rate of spontaneous mortality of the control and cOrai1 KO male mice housed under standard conditions was monitored throughout their lifespan over an observational period of 30 months. The results were analyzed according to Kaplan–Meier survival curve analysis. 

### 2.3. In Vivo Mouse Weight, Ex Vivo Adipose Tissue Weight, and Grip Strength Analyses

Whole body weight and grip strength were determined in the 4–6-month- and 10–14-month-old control and cOrai1 KO mice. After euthanasia, specific adipose depots (subcutaneous, epididymal, and retroperitoneal) were anatomically dissected and weighed. The tissue weights were normalized by dividing the absolute tissue weight by the body weight for each individual mouse and plotted as the percentage of body weight. The average amount of brown adipose tissue (BAT) was also evaluated and expressed as a percentage of the total adipose tissue (Supplementary Figure S1). The peak force produced by the mice during instinctive grasping (i.e., grip strength) was measured by holding the mice by the tails and lowering them onto metal grating connected to the shaft of a Shimpo Fgv 0.5× force transducer (Metrotec Group, Lezo, Spain). Once the mouse had firmly grabbed the grating, its tail was given a steady, gentle pull [49]. Measurements of the peak force generated by each mouse using both their fore- and hindlimbs were repeated three times with appropriate rest intervals (at least 30 s) to avoid fatigue. The highest value of peak force measured was recorded for each mouse.

### 2.4. The Treadmill Endurance Exercise Task

Ten-to eleven-month-old control and cOrai1 KO old mice were pre-trained on a 6-lane treadmill (Columbus Instruments, Columbus, OH, USA) at a modest treadmill speed of 5 m/min for 5 min at a 0° incline over 3 consecutive days. On the fourth day, the mice were subject to a 1 h endurance run on the treadmill (with a 700 m total distance), starting at 5 m/min for 10 min, followed by 25 min at a speed of 10 m/min, then 20 min at 15 m/min, and finally 5 min at 20 m/min. Continued running was encouraged by delivering brief (<1 s) sprays of air to the mouse’s backside using a Whoosh Duster™ (Control company, Houston, TX, USA). The number of rests during each 5 min window of time was recorded for each mouse. Exhaustion was defined as the inability of the mouse to re-engage on the treadmill after 3 consecutive <1 s sprays of air, as described previously [27]. The accumulative number of rests and the total running distance were recorded for each mouse.

### 2.5. Ex Vivo Muscle-Specific Force Measurements

Ex vivo assessments of muscle-specific force, the kinetics of force production/relaxation, and susceptibility to fatigue during repetitive high-frequency stimulation were made in the excised EDL muscles. Briefly, mice were anesthetized by intra-peritoneal injection of an anesthetic cocktail, as described previously [30]. EDL muscles from 10–11-month-old control and cOrai1 KO mice were isolated, tied using 4-0 surgical sutures, carefully excised, attached to a servo motor and force transducer (1200 A, Aurora Scientific, Aurora, ON, Canada), and placed between two platinum electrode plates in a chamber continuously perfused with oxygenated Ringer solution, containing 137 mM NaCl, 5 mM KCl, 1.2 mM NaH_2_PO_4_, 1 mM MgSO_4_, 2 mM CaCl_2_, 10 mM glucose, and 24 mM NaHCO_3_, pH = 7.4. Before starting each experiment, the optimal stimulation intensity and muscle length (*L_o_*) were determined using a series of 1 Hz twitch stimulation trains to guide stretching the muscle to the length that generated the maximal force (*F_o_*). After establishing *L_o_*, the muscles were first equilibrated using three 500 ms 150 Hz tetani delivered at 1 min intervals. The EDL muscles were then subjected to a force–frequency stimulation protocol (from 1 to 250 Hz for EDL muscles). To assess muscle fatigability, the EDL muscles were subjected to a repetitive, high-frequency stimulation protocol (60 stimulus trains of 50 Hz and 500 ms in duration delivered every 2.5 s). Muscle force was recorded using dynamic muscle control software v5.415 (Aurora Scientific, Aurora, ON, Canada) and analyzed using dynamic muscle analysis software v5.200 (Aurora Scientific, Aurora, ON, Canada). Muscle physiological CSA and specific force were calculated as described previously [50].

### 2.6. Sample Preparation for Histology and Electron Microscopy (EM)

The EDL muscles were quickly dissected from euthanized 4–6-month- and 10–14-month-old control and cOrai1 KO mice, pinned on Sylgard dishes, fixed at room temperature with 3.5% glutaraldehyde in 0.1 M NaCaCO buffer (pH 7.2), and stored in the fixative at 4 °C until embedding. The fixed muscles were then postfixed in osmium tetroxide (OsO_4_), stained en bloc, dehydrated, and embedded as previously described [51,52]. For histological examination by light microscopy, semithin sections (~700 nm) were cut using a Leica Ultracut R microtome (Leica Microsystem, Vienna, Austria) with a diamond knife (Diatome, Biel, Switzerland) and stained in a solution containing 1% toluidine blue O and 1% sodium borate (tetra) in distilled water for 3 min on a hot plate at 55–60 °C. After washing and drying them, the sections were mounted with DPX media for histology (Sigma–Aldrich, Milan, Italy) and observed with a Leica DMLB light microscope connected to a DFC450 camera equipped with Application Suite v 4.13.0 (Leica Microsystem, CMS GmbH, Vienna, Austria) for Windows 11 pro v 23H2 (Microsoft, Seattle, WA, USA). For the EM, ultrathin sections (~50 nm) were cut using an Ultracut R microtome (Leica Microsystem, Vienna, Austria) with a diamond knife (Diatome, Biel, Switzerland) and double-stained with a uranyl acetate replacement and lead citrate. The sections were viewed in an FP 505 Morgagni Series 268D electron microscope (FEI Company, Brno, Czechia) equipped with a Megaview III digital camera and the Soft Imaging System v 3.2 (Olympus Soft Imaging Solutions, Munster, Germany) at 60 kV.

### 2.7. Quantitative Analyses of the Histological and EM Images

Histological sections of the EDL muscles from the control and cOrai1 KO mice stained in a solution containing 1% toluidine blue O and 1% sodium borate (tetra) in distilled water were analyzed with a Leica DMLB light microscope connected to a DFC450 camera (Leica Microsystem, Vienna, Austria) equipped with Application Suite v 4.13.0 (Leica Microsystem, Vienna, Austria) for Windows 11 pro v 23H2 (Microsoft, Seattle, WA, USA) to quantify the following:

− *Muscle fiber CSA:* CSA was measured in light microscopy images from semithin transverse sections of whole EDL muscles from the 4–6-month- and 10–14-month-old control (*n* = 3–5) and cOrai1 KO (*n* = 3–5) mice by manually tracing the individual fibers, followed by automatic calculation of the fiber CSA with Application Suite software v 4.13.0 (Leica Microsystem, Vienna, Austria) for Windows 11 pro v 23H2 (Microsoft, Seattle, WA, USA).
− *Percentage of fibers with TAs:* For quantitative analyses of TAs, light microscopy images of histological semithin transverse sections of the whole EDL muscles from the 10–14-month-old control (*n* = 3) and cOrai1 KO (*n* = 3) mice were taken. The percentage of muscle fibers containing TAs was evaluated in 1756 EDL fibers from the control mice and 1300 EDL fibers from the cOrai1 KO mice by counting the number of fibers presenting areas within the sarcoplasm with abnormal dark stained material.

EM images of the EDL muscles fibers from the control and cOrai1 KO mice were taken with a FP 505 Morgagni Series 268D electron microscope (FEI Company, Brno, Czechia) equipped with a Megaview III digital camera and the Soft Imaging System v 3.2 (Olympus Soft Imaging Solutions, Munster, Germany) and used for the following quantitative analyses:

− *Mitochondrial number and damage*: The number of mitochondria/100 µm^2^ and the percentage of severely altered mitochondria were evaluated from electron micrographs of non-overlapping regions randomly collected from longitudinal sections at 14,000× magnification. For each specimen, 10 fibers and 5 micrographs/fiber were analyzed. The number of severely altered mitochondria, classified as previously described [51], was reported as a percentage value of the total number of mitochondria.
− *TA number/fiber and TA average size:* For quantitative analyses of the TA number/fiber and their average size, electron micrographs from transverse sections of whole EDL muscles collected from 10–14-month-old control (*n* = 3) and cOrai1 KO (*n* = 3) mice were taken.
− *Presence of SR stacks.* The percentage of fibers presenting SR stacks and the number of SR stacks in 100 µm^2^ were determined in electron micrographs of non-overlapping regions randomly collected from transverse sections at 28,000× magnification. For each specimen, 10–15 representative fibers and 5 micrographs/fiber were analyzed.
− *Total TT length at the I band.* Extensions of the TT network into the I band region of the sarcomere (i.e., total TT length) were measured in electron micrographs of non-overlapping regions randomly collected from transverse sections at 28,000× magnification and reported as length (µm)/100 µm^2^. The total TT network included both triadic TTs, identified as the emerging tubules between the two SR terminal cisternae in the transverse or longitudinal junctions, and non-triadic TTs, characterized by a narrow profile and lacking an electron-dense content. For each specimen, 10–15 representative fibers and 5 micrographs/fiber were analyzed. Quantitative analyses of both the SR stacks and TT network extensions were conducted using Analy-SIS software v 3.2 (Olympus Soft Imaging Solutions, Munster, Germany).

### 2.8. Statistical Analyses

Statistical significance was determined using either an unpaired, two-tailed Student’s *t*-test (when comparing means between two groups) or two-way ANOVA, followed by the post hoc Tukey test for repeated measures (when comparing across more than two groups) using Prism 9 software (GraphPad Software, Boston, MA, USA). The normal distribution of the data was checked with the Shapiro–Wilk normality test using Prism 9 software (GraphPad Software, Boston, MA, USA). When a Gaussian distribution was not confirmed, a non-parametric t-test (Mann–Whitney U test) was used. Survival data were evaluated by Kaplan–Meier analysis, and statistical significance was assessed using a Log-rank Mantel–Cox test. In all cases, differences were considered statistically significant at * *p* < 0.05. All the data are presented as means ± SEM.

## 3. Results

### 3.1. cOrai1 KO Mice Exhibit Reduced Survival

**Figure 1:**
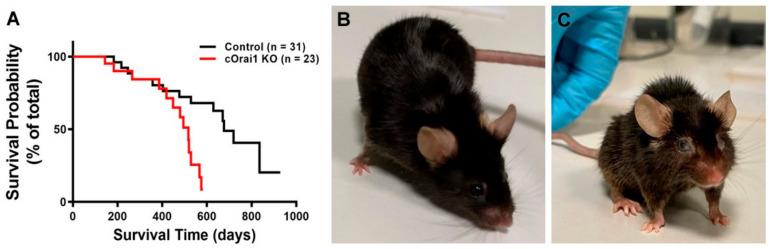
Reduced lifespan of cOrai1 KO and appearance of 14-month-old mice. (**A**) Kaplan–Meier survival curves of control and cOrai1 KO male mice; *p* < 0.01 as evaluated by Log rank (Mantel–Cox) test; *n* = number of animals. (**B**,**C**) Representative images of a control (**B**) and a cOrai1 KO mouse (**C**) at 14 months of age.

cOrai1 KO mice (i.e., mice specifically lacking the expression of the ORAI1 Ca^2+^ release-activated Ca^2+^ (CRAC) channel in the skeletal muscle) were generated using the cre-loxP system (crossing *Orai1*-floxed mice with MCK-cre mice) in the laboratory of Robert T. Dirksen (the University of Rochester), as described previously [27]. *Orai1*-floxed mice lacking Cre-recombinase were used as the controls. The rate of spontaneous mortality of the control and cOrai1 KO mice under standard housing conditions was determined during an observational period of 30 months. The survival curves of the control and cOrai1 KO mice are shown in Figure 1A. Interestingly, the mortality rate of the cOrai1 KO mice (50% survival < 500 days) was significantly higher (*p* < 0.01) than that of the control mice (50% survival < 700 days). Of note, no cOrai1 KO mouse survived beyond 600 days, while some of the control mice survived for up to 800 days. Moreover, the appearance of the cOrai1 KO mice at 14 months of age was visibly different from that of the age-matched control mice, including frizzy and faded fur, a smaller size and ocular opacity, hunched backs in a few cases, etc. (Figure 1B,C).

### 3.2. cOrai1 KO Mice Exhibit a Reduced Body Weight, EDL Mass and Cross-Sectional Area (CSA), and Grip Strength

**Figure 2:**
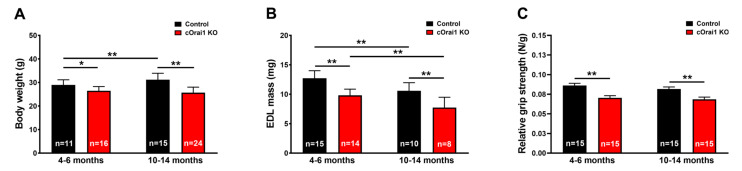
Reduced weight, muscle mass, and grip strength of young and older cOrai1 KO mice. (**A**) Average body weight of control and cOrai1 KO male mice at 4–6 months of age (**left**) and 10–14 months of age (**right**). (**B**) Average EDL muscle mass of control and cOrai1 KO male mice at 4–6 months of age (**left**) and 10–14 months of age (**right**). (**C**) Relative grip strength (normalized to body weight) of control and cOrai1 KO male mice at 4–6 months of age (**left**) and 10–14 months of age (**right**). Data are shown as means ± SEM. * *p* < 0.05 and ** *p* < 0.01 as evaluated by two-way ANOVA, followed by post hoc Tukey’s multiple-comparisons test. In panels (**A**,**C**), *n* = number of mice; in panel (**B**), *n* = number of EDL muscles.

**Figure 3:**
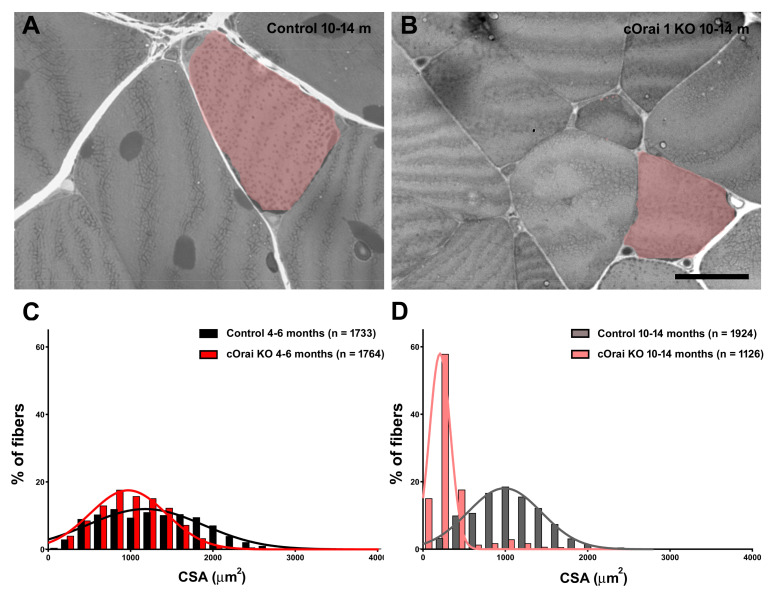
Fiber atrophy in EDL muscles of cOrai1 KO mice. (**A**,**B**) Representative histological images of transverse sections from EDL muscles of 10–14-month-old male control (**A**) and cOrai1 KO (**B**) mice: false labeling in pink marks fibers with evidently different sizes between the two genotypes. (**C**,**D**) Distribution frequency of muscle fiber CSA in EDL muscles from 4–6-month-old (**C**) and 10–14-month-old (**D**) male control and cOrai1 KO mice. Data are shown as mean values. Scale bar shown in panel (**B**) applies to both panels (**A**,**B**): 20 µm. In panels (**C**,**D**), *n* = number fibers analyzed from 3–5 mice.

We measured body weight, EDL mass, and grip strength in young adult (4–6 months) and older adult (10–14 months) control and cOrai1 KO mice (Figure 2). Similar to the results reported previously for younger cOrai1 KO mice [27], the average body weight (Figure 2A) and EDL mass (Figure 2B) were significantly reduced in the 4–6- and 10–14-month-old cOrai1 KO mice; thus, our results confirm the prior findings but across two distinct age ranges (4–6 and 10–14 months of age). This reduction in body weight between the two groups of mice was more pronounced at 10–14 months of age, as the control mice exhibited an increase in body weight between 4–6 and 10–14 months of age that was absent in the cOrai1 KO mice (Figure 2A). Moreover, different adipose deposits (e.g., cervical, interscapular, axillary–thoracic, mesenteric, abdominal–pelvic, retroperitoneal, inguinal) were anatomically dissected (Supplementary Figure S1) and weighed for both groups of mice at 4–6 (Supplementary Figure S1A,B) and 10–14 months of age and reported as percentages of total body fat (Supplementary Figure S1C). The brown adipose tissue (BAT) mass was also evaluated (Supplementary Figure S1D). The percentages of total body fat and BAT were unchanged between the 4–6-month-old control and cOrai1 KO mice. The total body fat content was increased in the 10–14-month-old mice despite a reduction in their body weight, suggesting the role of a reduction in muscle mass. Finally, BAT seemed to be differently affected in the two groups of mice at the older age (Supplementary Figure S1D). Carrell et al. [27] found that a reduction in the EDL mass of young adult cOrai1 KO mice was due in part to a modest reduction in the fiber CSA. Thus, we assessed the fiber CSA in histological images taken from transverse sections of the EDL muscles from both the 4–6-month- and 10–14-month-old control and cOrai1 KO mice (Figure 3). Consistent with prior results, analysis of the distribution frequency of the myofiber CSA revealed a modest shift to smaller areas in the cOrai1 KO mice at 4–6 months of age (Figure 3C). This shift to lower myofiber CSA values was even greater in the 10–14-month-old cOrai1 KO mice (Figure 3D), a feature that can explain the significantly reduced EDL mass observed in the cOrai1 KO mice at 10–14 months of age when compared to that observed at 4–6 months of age (see Figure 2B). 

Moreover, consistent with the observed reduction in muscle mass (Figure 2B), the in vivo grip strength (normalized to body weight) was also significantly reduced in both the 4–6-month-old and 10–14-month-old cOrai1 KO mice (Figure 2C).

### 3.3. Ten- to Eleven-Month-Old cOrai1 KO Mice Exhibit Impaired In Vivo and Ex Vivo Skeletal Muscle Function

**Figure 4:**
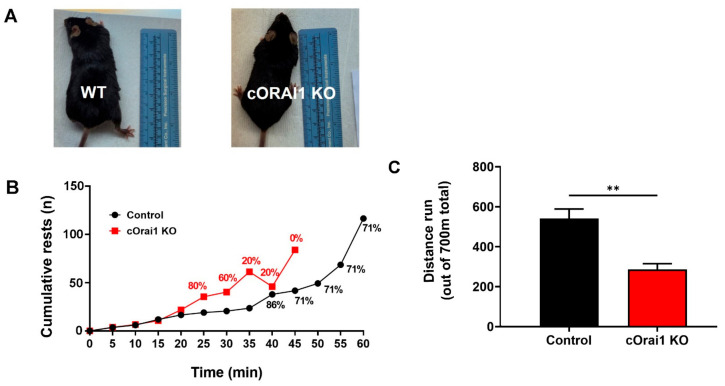
cOrai1 KO mice exhibit reduced in vivo exercise tolerance. (**A**) Representative images of 10.5-month-old control and cOrai1 KO mice. (**B**) Cumulative rests during a treadmill run endurance test (1 h total duration, 700 m total distance, slowly increasing speed, flat/no elevation). Percentages indicate the relative number of animals that continued to run on the treadmill after this time point. (**C**) Total distance run (700 m maximum). Data are shown as means ± SEM. ** *p* < 0.01 as evaluated by unpaired, two-tailed Student’s *t*-test, *n* = seven control mice (three males and four females) and *n* = five cOrai1 KO mice (three males and two females).

**Figure 5:**
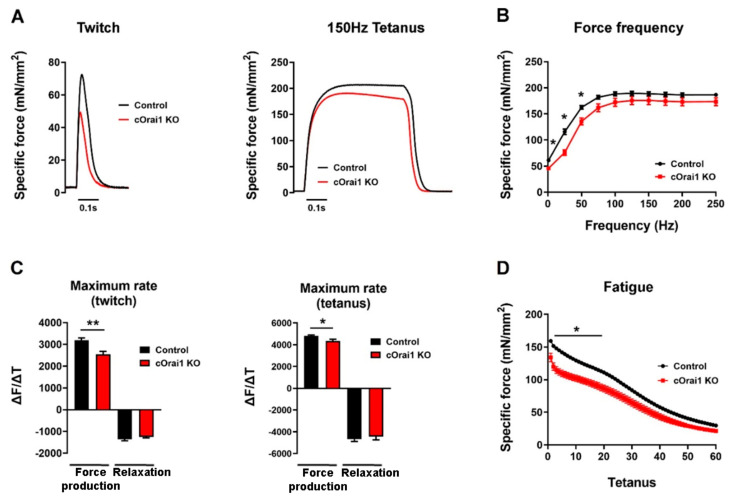
cOrai1 KO mice exhibit impaired ex vivo EDL-specific force production and contraction kinetics. (**A**) Example twitch (**left**) and tetanic (**right**) -specific force (mN/mm^2^) traces in EDL muscles from 10.5-month-old control and cOrai1 KO mice. (**B**) Specific force versus frequency (1–250 Hz) relationship. (**C**) Maximum rate of EDL force production and relaxation for twitch (**left**) and tetanic (**right**) stimulation (∆F(mN/mm^2^)/∆t(second)). (**D**) Peak EDL-specific force during repetitive, high-frequency stimulation (60 stimulus trains, 500 ms duration trains, 50 Hz stimulation frequency, delivered every 2.5 s). Data are shown as means ± SEM. * *p* < 0.05 and ** *p* < 0.01 as evaluated by unpaired Student’s *t*-test, *n* = 12 muscles from seven control mice (three males and four females) and *n* = 9 muscles from five cOrai1 KO mice (three males and two females).

We compared the in vivo exercise endurance (Figure 4) and ex vivo EDL contractile function (Figure 5) of the 10–11-month-old control and cOrai1 KO mice (images of representative 10.5-month-old mice are shown in Figure 4A), an age just prior to the increased incidence of spontaneous death observed for the cOrai1 KO mice (see Figure 1A). In vivo exercise endurance was evaluated using a treadmill endurance run task in which the mice were encouraged to run for 1 h on a flat (0°) treadmill with a slowly increasing speed (see Materials and Methods for details). The total distance traveled was 700 m for the mice that completed the task. The 10–11-month-old control mice typically rested often, and ~30% refused to run at some point during the 1 h task (Figure 4B, black). Meanwhile, the 10–11-month-old cOrai1 KO mice rested even more often, and none of these mice completed the entire 1 h task (Figure 4B, red). As a result, the cOrai1 KO mice exhibited a significant reduction in their average total distance run during the task, consistent with greater exercise intolerance.

Since performance in behavioral assays such as the treadmill endurance task is subject to multiple variables in addition to intrinsic muscle function (e.g., mouse weight, motivation, motor unit complexity, synaptic transmission), we also compared the ex vivo contractile function of isolated EDL muscles from these same 10–11-month-old control (*n* = 7) and cOrai1 KO (*n* = 5) mice (Figure 5), which is a finding not measured or reported previously. The maximal magnitude of the electrically evoked specific force was reduced in the EDL muscles from the 10–11-month-old cOrai1 KO mice compared to that of the age-matched control mice (Figure 5A,B). A reduction in the peak specific force was observed across all stimulation frequencies, though statistical significance in this limited cohort was only observed at the lower stimulation frequencies. In addition, the maximal rate of specific force production, but not the maximum rate of relaxation, during both twitch and tetanic (150 Hz) stimulation was significantly reduced in the EDL muscles from the 10–11-month-old cOrai1 KO mice (Figure 5C). Finally, peak specific force production during repetitive, high-frequency stimulation was also significantly reduced in the EDL muscles from the 10–11-month-old cOrai1 KO mice (Figure 5D). Together, the results in Figure 4 and Figure 5 demonstrate that skeletal muscle function is significantly compromised in cOrai1 KO mice at an age immediately prior to the observed increase in mortality compared to that of the control mice.

### 3.4. Increased Percentage of Damaged Mitochondria in EDL Fibers from cOrai1 KO Mice

**Figure 6:**
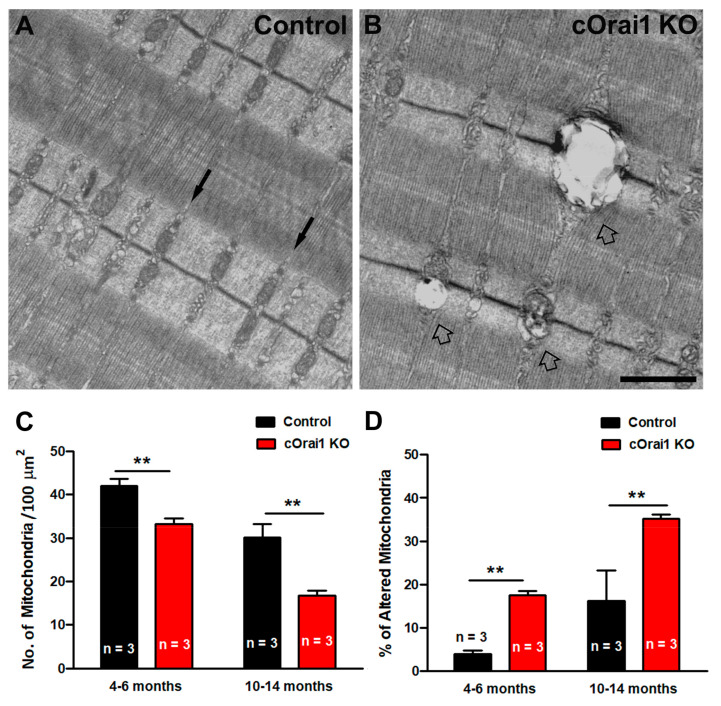
Mitochondrial damage is increased in EDL muscles of cOrai1 KO mice. (**A**,**B**) Representative EM images of longitudinal sections of EDL muscle fibers from 4–6-month-old male control (**A**) and cOrai1 KO (**B**) mice. Black arrows in (**A**) point to inter-myofibrillar mitochondria, placed in the correct I band position in control mice, while empty arrows in (**B**) point to damaged mitochondria in cOrai1 KO mice. (**C**,**D**) Bar plots showing the number of mitochondria/area (**C**) and percentage of altered mitochondria (**D**) in 4–6- and 10–14-month-old control and cOrai1 KO mice. Data are shown as means ± SEM; ** *p* < 0.01 as evaluated by two-tailed, unpaired Student´s *t*-test. Scale bar in panel (**B**) applies to both panels (**A**,**B**): 1 µm. *n* = number of mice.

Mitochondrial loss, damage, and mislocalization are phenomena that are widely described in the literature, both in aging and in muscle diseases [51,52,53,54,55,56,57,58,59,60]. Thus, we quantified both the total number of mitochondria per 100 µm^2^ and the percentage of damaged mitochondria in EM images of EDL muscle fibers from the 4–6- and 10–14-month-old control and cOrai1 KO mice (Figure 6). Most of the mitochondria in the EDL fibers of the control mice were not damaged, as they exhibited a dark matrix, with the inner and outer membranes clearly visible, and they were properly positioned within the I band of the sarcomere (Figure 6A). On the other hand, in some areas of the fibers from the cOrai1 KO animals, the mitochondrial ultrastructure was compromised (Figure 6B). Quantitative analyses revealed that the total number of mitochondria per 100 µm^2^ was significantly reduced in the EDL muscles of the cOrai1 KO mice (Figure 6C), while the percentage of damaged mitochondria was significantly increased (Figure 6D), both at 4–6 months and 10–14 months of age, compared to that observed for age-matched control samples. 

### 3.5. Tubular Aggregates (TAs) Do Not Form in EDL Muscles of Aging cOrai1 KO Mice

**Figure 7:**
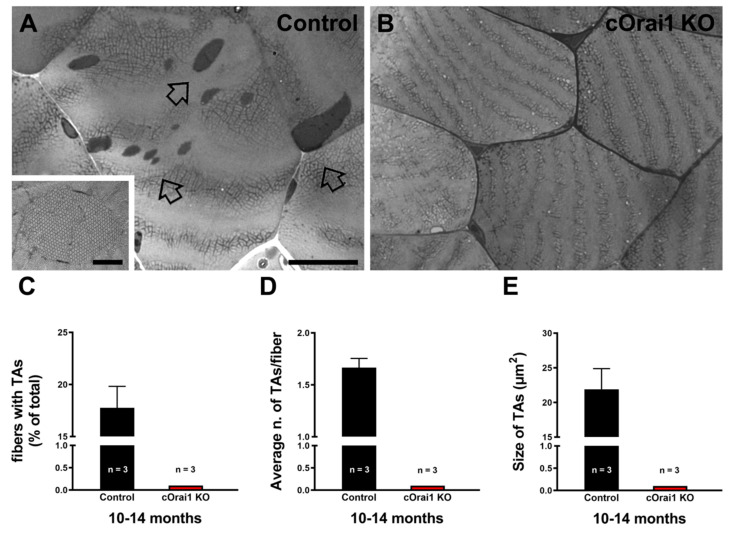
The incidence of TAs is reduced in EDL muscles of cOrai1 KO mice. (**A**,**B**) Representative histological images from transverse sections of EDL muscles of 10–14-month-old male control (**A**) and cOrai1 KO (**B**) mice: empty arrows point to TAs. (**C**–**E**) Bar plots showing the percentage of EDL fibers containing TAs (**C**), average number of TAs per fiber (**D**), and average TA size (**E**). Data are shown as means ± SEM. Scale bar shown in panel (**A**) applies to both panels (**A**,**B**): 0.5 µm; scale bar in inset: 0.1 µm. *n* = number of EDL muscles.

TAs are tightly packed straight tubes of SR membrane origin (see enlarged details in Figure 7A). TAs represent an age-related remodeling of the SR in the muscle fibers of male mice that is associated with dysfunction in SOCE [26,45]. TAs are also the main histopathological hallmark in the skeletal muscle fibers of patients affected by TAM, a disease associated with gain-of-function mutations in the human *STIM1* and *ORAI1* genes [34,35,36,37,38,39,40,41] and, more recently, with some mutations in the *CASQ1* and *RYR1* genes [34,42,43]. Here, we quantified the presence of TAs in histological transverse sections of the EDL muscles from 10–14-month-old control and cOrai1 KO male mice. Surprisingly, TAs were only found in EDL muscles from the control mice (Figure 7A, empty arrows) but not in samples from the cOrai1 KO mice (Figure 7B). Specifically, when they were present, we quantified the (i) percentage of fibers with TAs (Figure 7C); (ii) the average number of TAs per fiber (Figure 7D); and (iii) the average TA size (Figure 7E). The data obtained from these quantitative analyses of the TAs in the control mice and the evidence of their absence in the cOrai1 KO mice suggest that ORAI1 is crucial to the formation of TAs during aging.

### 3.6. EDL Fibers from cOrai1 KO Mice Exhibit an Increase in SR Stacks without TT Extensions

**Figure 8:**
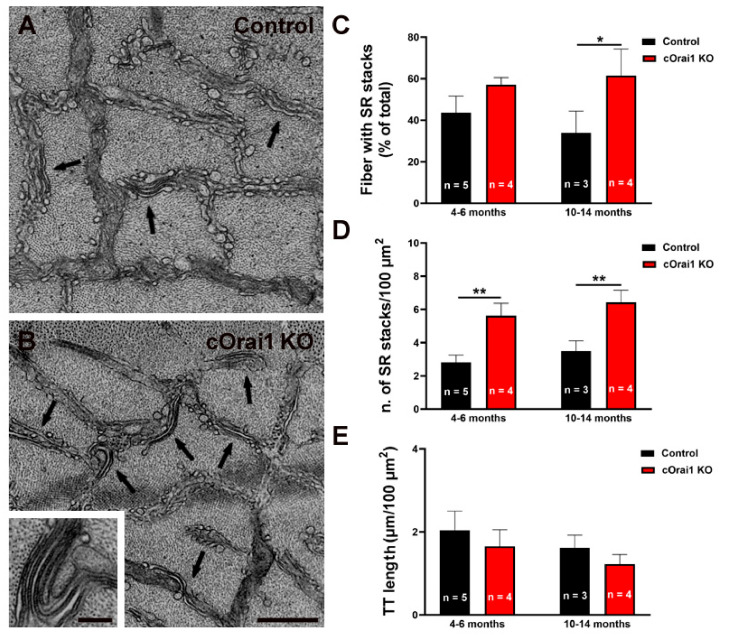
SR stacks are increased in EDL fibers of cOrai1 KO mice. (**A**,**B**) Representative EM images of EDL muscles in transverse sections from 4–6-month-old male control (**A**) and cOrai1 KO (**B**) mice; arrows point to SR stacks; inset in panel (**B**) shows an SR stack at higher magnification. (**C**–**E**) Bar plots summarizing the percent of fibers with SR stacks (**C**), number of SR stacks/100 µm^2^ (**D**), and TT length (**E**). Data in (**C**–**E**) are shown as means ± SEM. * *p* < 0.05 and ** *p* < 0.01 as evaluated by two-way ANOVA, followed by post hoc Tukey’s multiple-comparisons test. Scale bar shown in panel (**B**) applies to both panels (**A**,**B**): 0.5 µm; scale bar in inset: 0.1 µm. *n* = number of EDL muscles.

We previously demonstrated that exercise induces a remodeling of the SR to form flat parallel stacks in association with extensions of the TTs in the I band, which results in the formation of SR-TT junctions [22]. These SR-TT junctions within the I band are referred to as CEUs since they are associated with an increase in STIM1-ORAI1 co-localization [22], SOCE activity, SR Ca^2+^ store refilling, Ca^2+^ release, and force production during repetitive stimulation [23,24]. Here, we quantified the SR stacks and TTs in the I band in EM images of transverse sections from the EDL muscles of both young (4–6 months) and older adult (10–14 months) control (Figure 8A) and cOrai1 KO (Figure 8B) mice. These analyses revealed an increase in the percentage of fibers presenting SR stacks in the EDL fibers from the 10–14-month-old cOrai1 KO mice when compared to those of the age-matched control mice, while no such differences were observed in the EDL muscles from the younger mice (Figure 8C). In contrast, the number of SR stacks per unit area (100 µm^2^) was significantly increased in the EDL muscles at both ages (Figure 8D). We also quantified the total TT length within the I band, which is the second element required for a functional CEU. This analysis did not reveal any statistically significant change in the TT length in the I band in the EDL muscles of the cOrai1 KO mice (Figure 8E). The increased presence of SR stacks in the I band without a corresponding increase in associated TTs indicates that the number of functional CEUs was not increased in the EDL muscles of the cOrai1 KO mice.

## 4. Discussion

### 4.1. Main Findings

In a previous publication, cOrai1 KO mice were studied from 3 to 12 months of age, with most of the mice being 3 to 6 months old [27]. In order to assess the potential long-term effects of *Orai1* ablation, here, we compared results obtained in cOrai1 KO mice across two age ranges: 4–6 months and 10–14 months of age. The older group range (i.e., 10–14 months of age) was chosen based on the reduced survival of the cOrai1 KO mice compared to that of the control mice (Figure 1A). During this period, the cOrai1 KO mice exhibit a reduced body weight, skeletal muscle mass, and grip strength (Figure 2), as well as a decreased fiber CSA (Figure 3), exercise intolerance (Figure 4), and reduced EDL-specific force production (Figure 5). We also detected increased mitochondrial damage (Figure 6), an alteration that could contribute to muscle dysfunction and that is commonly found in aging and several muscle disorders [51,53,56,58,59,60]. Overall, these results are consistent with, but more severe than, those previously reported for a younger mouse cohort [27]. Thus, *Orai1* ablation results in a slowly progressive and increasing impairment of muscle function, consistent with ORAI1-dependent SOCE not only being important for muscle growth [11,27,30] but also for maintenance of muscle mass, force production, and exercise tolerance later in life.

Importantly, we also found that (a) EDL muscle fibers from aging cOrai1 KO mice do not develop TAs, as muscle fibers from age-matched control mice do (Figure 7), and (b) an increased number of SR stacks at both 4–6 months and 10–14 months of age (Figure 8), which, however, was not accompanied by elongation of the TTs in the I band, the second element required for full assembly of functional CEUs (Figure 8). 

### 4.2. Our Present Results in Relation to Previous Findings

We previously suggested that the deficits in maximal force generation and exercise endurance observed in cOrai1 KO mice are due primarily to a reduced oxidative fatigue-resistant fiber content and calcium store capacity, while acute (1-month) deletion of *Orai1* in adult muscle (using tamoxifen-inducible, muscle-specific *Orai1* KO mice generated by crossing *Orai1*-floxed mice with muscle-specific, inducible human skeletal actin (HSA)-mutated estrogen receptor (Mer)-Cre-Mer (MCM) mice) did not significantly alter the muscle fiber type distribution, CSA, or force production [27]. However, Michelucci et al. [23] subsequently reported a marked reduction in sustained force production during repetitive, high-frequency stimulation in EDL muscles after the acute knockout of *Orai1* in adult mice sufficient to abolish SOCE. Thus, ORAI1 function is clearly important for sustained muscle force production during repetitive stimulation. Unfortunately, neither our study nor Michelucci et al. [23] investigated the impact of long-term *Orai1* knockout in adult mice (e.g., from 4 months of age to 12 months of age). Thus, the effect of long-term, tamoxifen-induced *Orai1* knockout in adult mice on sustained muscle force production during repetitive, high-frequency stimulation and the age-dependent assembly of CEUs and formation of TAs remain unknown. 

### 4.3. Lack of Orai1 Prevents the Assembly of Tubular Aggregates (TAs)

An important new finding of this study is the absence of TAs in the EDL muscles of 10–14-month-old male cOrai1 KO mice (Figure 7). TAs are peculiar aggregations of straight SR tubes found in muscle biopsies of patients with TAM, which are linked to gain-of-function mutations in *ORAI1* and *STIM1* [34,35,36,37,38,39,40,41,47]. The development of TAs resulting from mutations in *ORAI1* and *STIM1* could be the result of excessive constitutive Ca^2+^ entry, which could lead to chronically elevated myoplasmic Ca^2+^ levels. In this scenario, TAs may reflect a compensation for the inability of the SR/plasma membrane to clear excessive Ca^2+^. In addition, TAs are also observed in fast-twitch muscle fibers from aged male mice [26,45,46]. Some authors have suggested that with age, RYRs becomes leaky [61], and TAs serve as an additional Ca^2+^ buffer in cases of excessive Ca^2+^ accumulation in the cytoplasm. The addition of voluntary wheel running exercise prevents the formation of TAs in male mice during aging [26], possibly by limiting age-dependent enhancement of RYR Ca^2+^ leakage, as observed in sedentary mice.

The results presented in this study suggest that ORAI1-dependent Ca^2+^ entry is required for the formation of TAs in male mice during the aging process. This finding supports the idea that TAs may serve as an adaptive mechanism of the skeletal muscle designed to limit the damaging effects of excessive accumulation of Ca^2+^ in the myoplasm during aging and TAM (due to either excessive Ca^2+^ entry or SR leakage).

### 4.4. Lack of ORAI1 Results in Incomplete Assembly of Ca^2+^ Entry Units (CEUs)

The intracellular sites of STIM1-ORAI1 interaction in the skeletal fibers remained elusive for many years after SOCE was first identified in adult muscle [62,63]. However, experimental evidence collected over the past decade suggests that (i) STIM1 is mainly enriched in the SR present in the I band [22,30], (ii) ORAI1 is present within the TTs [22,30], and (iii) STIM1-ORAI1 co-localization in the I band increases during exercise due to the translocation of ORAI1-containing TTs from the triad to the I band [22]. Increased interaction between STIM1 and ORAI1 in the I band reflects the formation of CEUs, dynamic junctions between SR stacks and extended TTs [22]. Here, we found the incidence of SR stacks to be increased in the EDL muscles of the cOrai1 KO mice (Figure 8), though without associated TTs. The observation that this increase in SR stacks is not accompanied by extensions of the TTs indicates incomplete assembly of the CEUs and is consistent with the absence of ORAI1-dependent Ca^2+^ entry into the muscle fibers of cOrai1 KO mice [27]. The detailed molecular mechanisms that underlie the increased assembly of SR stacks in the skeletal muscle of cORAI1 KO mice are unclear and deserve further investigation. We know that the total releasable Ca^2+^ store content is significantly reduced in muscle fibers from adult cOrai1 KO mice but not in younger adolescent (4–6-week-old) cOrai1 KO mice (see Figure 1H in [27]). Hence, the increased presence of SR stacks in older cOrai1 KO mice could represent a futile attempt of the fibers to enhance Ca^2+^ entry and store content, which is doomed to fail due to the constitutive lack of ORAI1. As it has been shown that STIM1 interacts with and modulates the function of SERCA pumps [64], the formation of SR stacks may also reflect an attempt to augment SERCA function in order to sequester cytoplasmic Ca^2+^ better in older muscle fibers.

### 4.5. Lifespan Reduction and Premature Aging in Mice Lacking ORAI1

Aging is a physiological process in which cell division and tissue repair are not as efficient as in adulthood [65,66,67]. In skeletal muscle, aging is characterized by a loss of muscle mass (sarcopenia), reduced specific force production, and enhanced susceptibility to fatigue. Loss of muscle mass likely involves a combination of reduced fiber CSAs and the loss of fast-twitch motor units, with some being cross-innervated with slow-twitch motor neurons [68,69]. Ca^2+^ plays a crucial role as a second messenger in many skeletal muscle functions, including growth, contraction, and gene transduction. The lack of ORAI1-mediated Ca^2+^ entry into the skeletal muscle of cOrai1 KO mice alters muscle growth and fiber type determination [27]. Some of the changes observed in the muscles of 10–14-month-old cOrai1 KO mice resemble a premature aging phenotype [51,52], including reduced muscle mass (Figure 2), decreased specific force production (Figure 5), and increased mitochondrial damage (Figure 6), as does their visual appearance at the end stage (i.e., smaller body size, eye opacity, frizzy and faded fur, hunched backs in a few cases, etc.) (Figure 1C). In contrast, other findings are not consistent with a premature aging phenotype [22,26,45], including an increased incidence of SR stacks (Figure 8) and a reduction in TAs (Figure 7). These age-dependent changes could reflect muscle adaptations designed to counteract the lack of ORAI1-dependent SOCE, possibly to limit the degree of premature muscle aging. In any event, the decreased lifespan and reduced muscle mass/function of cOrai1 KO mice clearly indicate that ORAI1-dependent SOCE plays an important role in muscle maintenance throughout life. 

## 5. Conclusions

SOCE was first measured in adult skeletal muscle in 2001 and was subsequently shown to be coordinated by STIM1 and ORAI1 in 2008 [10,11]. The first mutations in *STIM1* and *ORAI1* linked to TAM were identified several years later [35,36,39]. Finally, intracellular sites of exercise-induced interactions between ORAI1 and STIM1 in skeletal muscle fibers (i.e., CEUs) were first described in 2017 [22]. Nevertheless, a comprehensive understanding of STIM1-ORAI1 SOCE and its role in skeletal muscle health and disease have yet to be fully elucidated. Here, we show that muscle-specific *Orai1* ablation in mice leads to a progressive impairment of skeletal muscle function that ultimately leads to a reduced lifespan. 

The overall picture emerging from this work is that ORAI1 function is important for skeletal muscle growth, force production, exercise tolerance, and long-term maintenance of muscle mass. Importantly, we also show that the absence of *Orai1* results in (a) absence of the age-related aggregation of TAs, consistent with TAs being formed as a result of excessive ORAI1-dependent Ca^2+^ entry, and (b) incomplete assembly of the CEUs, i.e., the TTs do not elongate to create contact with SR stacks, suggesting that ORAI1 is required for the elongation of the TTs and the formation of junctions with SR stacks in the I band. One interesting point that would be crucial to address in future experiments is whether the acute deletion of *Orai1* in adult muscle (instead of constitutive knockout) would impact age-related TA formation and CEU assembly.

## Data Availability

The original contributions presented in the study are included in the article/Supplementary Material, further inquiries can be directed to the corresponding author.

## Supplementary Materials

The following supporting information can be downloaded at: https://www.mdpi.com/article/10.3390/biomedicines12081651/s1, Figure S1: Adipose tissue in cOrai1 KO mice.

## Abbreviations

Ca^2+^, calcium ions; CEU, Ca^2+^ entry unit; CSA, cross-sectional area; EDL, extensor digitorum longus; EC coupling, excitation–contraction coupling; EM, electron microscopy; KO, knockout; SOCE, store operated Ca^2+^ entry; SR, sarcoplasmic reticulum; STIM1, stromal interaction molecule 1; TAM, tubular aggregate myopathy; TT, transverse tubule.

## References

[1] Calderon J.C., Bolanos P., Caputo C. (2014). The excitation-contraction coupling mechanism in skeletal muscle. Biophys. Rev..

[2] Dulhunty A.F., Banyard M.R., Medveczky C.J. (1987). Distribution of calcium ATPase in the sarcoplasmic reticulum of fast- and slow-twitch muscles determined with monoclonal antibodies. J. Membr. Biol..

[3] Hasselbach W. (1964). Relaxation and the Sarcotubular Calcium Pump. Fed. Proc..

[4] Hasselbach W., Oetliker H. (1983). Energetics and electrogenicity of the sarcoplasmic reticulum calcium pump. Annu. Rev. Physiol..

[5] Toyoshima C. (2009). How Ca^2+^-ATPase pumps ions across the sarcoplasmic reticulum membrane. Biochim. Biophys. Acta.

[6] Weber A. (1966). Energized Calcium Transport and Relaxing Factors. Curr. Top. Bioenerg..

[7] Dirksen R.T. (2009). Checking your SOCCs and feet: The molecular mechanisms of Ca^2+^ entry in skeletal muscle. J. Physiol..

[8] Lamb G.D. (2002). Excitation-contraction coupling and fatigue mechanisms in skeletal muscle: Studies with mechanically skinned fibres. J. Muscle Res. Cell Motil..

[9] Gissel H., Clausen T. (1999). Excitation-induced Ca^2+^ uptake in rat skeletal muscle. Am. J. Physiol..

[10] Lyfenko A.D., Dirksen R.T. (2008). Differential dependence of store-operated and excitation-coupled Ca^2+^ entry in skeletal muscle on STIM1 and Orai1. J. Physiol..

[11] Stiber J., Hawkins A., Zhang Z.S., Wang S., Burch J., Graham V., Ward C.C., Seth M., Finch E., Malouf N. (2008). STIM1 signalling controls store-operated calcium entry required for development and contractile function in skeletal muscle. Nat. Cell Biol..

[12] Liou J., Kim M.L., Heo W.D., Jones J.T., Myers J.W., Ferrell J.E., Meyer T. (2005). STIM is a Ca^2+^ sensor essential for Ca^2+^-store-depletion-triggered Ca^2+^ influx. Curr. Biol..

[13] Roos J., DiGregorio P.J., Yeromin A.V., Ohlsen K., Lioudyno M., Zhang S., Safrina O., Kozak J.A., Wagner S.L., Cahalan M.D. (2005). STIM1, an essential and conserved component of store-operated Ca^2+^ channel function. J. Cell Biol..

[14] Zhang S.L., Yu Y., Roos J., Kozak J.A., Deerinck T.J., Ellisman M.H., Stauderman K.A., Cahalan M.D. (2005). STIM1 is a Ca^2+^ sensor that activates CRAC channels and migrates from the Ca^2+^ store to the plasma membrane. Nature.

[15] Feske S., Gwack Y., Prakriya M., Srikanth S., Puppel S.H., Tanasa B., Hogan P.G., Lewis R.S., Daly M., Rao A. (2006). A mutation in Orai1 causes immune deficiency by abrogating CRAC channel function. Nature.

[16] Prakriya M., Feske S., Gwack Y., Srikanth S., Rao A., Hogan P.G. (2006). Orai1 is an essential pore subunit of the CRAC channel. Nature.

[17] Vig M., Peinelt C., Beck A., Koomoa D.L., Rabah D., Koblan-Huberson M., Kraft S., Turner H., Fleig A., Penner R. (2006). CRACM1 is a plasma membrane protein essential for store-operated Ca^2+^ entry. Science.

[18] Yeromin A.V., Zhang S.L., Jiang W., Yu Y., Safrina O., Cahalan M.D. (2006). Molecular identification of the CRAC channel by altered ion selectivity in a mutant of Orai. Nature.

[19] Stathopulos P.B., Zheng L., Li G.Y., Plevin M.J., Ikura M. (2008). Structural and mechanistic insights into STIM1-mediated initiation of store-operated calcium entry. Cell.

[20] Michelucci A., Garcia-Castaneda M., Boncompagni S., Dirksen R.T. (2018). Role of STIM1/ORAI1-mediated store-operated Ca^2+^ entry in skeletal muscle physiology and disease. Cell Calcium.

[21] Qiu R., Lewis R.S. (2019). Structural features of STIM and Orai underlying store-operated calcium entry. Curr. Opin. Cell Biol..

[22] Boncompagni S., Michelucci A., Pietrangelo L., Dirksen R.T., Protasi F. (2017). Exercise-dependent formation of new junctions that promote STIM1-Orai1 assembly in skeletal muscle. Sci. Rep..

[23] Michelucci A., Boncompagni S., Pietrangelo L., Garcia-Castaneda M., Takano T., Malik S., Dirksen R.T., Protasi F. (2019). Transverse tubule remodeling enhances Orai1-dependent Ca^2+^ entry in skeletal muscle. Elife.

[24] Michelucci A., Boncompagni S., Pietrangelo L., Takano T., Protasi F., Dirksen R.T. (2020). Pre-assembled Ca^2+^ entry units and constitutively active Ca^2+^ entry in skeletal muscle of calsequestrin-1 knockout mice. J. Gen. Physiol..

[25] Canato M., Scorzeto M., Giacomello M., Protasi F., Reggiani C., Stienen G.J. (2010). Massive alterations of sarcoplasmic reticulum free calcium in skeletal muscle fibers lacking calsequestrin revealed by a genetically encoded probe. Proc. Natl. Acad. Sci. USA.

[26] Boncompagni S., Pecorai C., Michelucci A., Pietrangelo L., Protasi F. (2021). Long-Term Exercise Reduces Formation of Tubular Aggregates and Promotes Maintenance of Ca^2+^ Entry Units in Aged Muscle. Front. Physiol..

[27] Carrell E.M., Coppola A.R., McBride H.J., Dirksen R.T. (2016). Orai1 enhances muscle endurance by promoting fatigue-resistant type I fiber content but not through acute store-operated Ca^2+^ entry. FASEB J..

[28] Darbellay B., Arnaudeau S., Konig S., Jousset H., Bader C., Demaurex N., Bernheim L. (2009). STIM1- and Orai1-dependent store-operated calcium entry regulates human myoblast differentiation. J. Biol. Chem..

[29] Thornton A.M., Zhao X., Weisleder N., Brotto L.S., Bougoin S., Nosek T.M., Reid M., Hardin B., Pan Z., Ma J. (2011). Store-operated Ca^2+^ entry (SOCE) contributes to normal skeletal muscle contractility in young but not in aged skeletal muscle. Aging.

[30] Wei-Lapierre L., Carrell E.M., Boncompagni S., Protasi F., Dirksen R.T. (2013). Orai1-dependent calcium entry promotes skeletal muscle growth and limits fatigue. Nat. Commun..

[31] Brotto M. (2011). Aging, sarcopenia and store-operated calcium entry: A common link?. Cell Cycle.

[32] Zhao B., Benson E.K., Qiao R., Wang X., Kim S., Manfredi J.J., Lee S.W., Aaronson S.A. (2009). Cellular senescence and organismal ageing in the absence of p21(CIP1/WAF1) in ku80^−/−^ mice. EMBO Rep..

[33] Garcia-Castaneda M., Michelucci A., Zhao N., Malik S., Dirksen R.T. (2022). Postdevelopmental knockout of Orai1 improves muscle pathology in a mouse model of Duchenne muscular dystrophy. J. Gen. Physiol..

[34] Bohm J., Bulla M., Urquhart J.E., Malfatti E., Williams S.G., O’Sullivan J., Szlauer A., Koch C., Baranello G., Mora M. (2017). ORAI1 Mutations with Distinct Channel Gating Defects in Tubular Aggregate Myopathy. Hum. Mutat..

[35] Bohm J., Chevessier F., Koch C., Peche G.A., Mora M., Morandi L., Pasanisi B., Moroni I., Tasca G., Fattori F. (2014). Clinical, histological and genetic characterisation of patients with tubular aggregate myopathy caused by mutations in STIM1. J. Med. Genet..

[36] Bohm J., Chevessier F., Maues De Paula A., Koch C., Attarian S., Feger C., Hantai D., Laforet P., Ghorab K., Vallat J.M. (2013). Constitutive activation of the calcium sensor STIM1 causes tubular-aggregate myopathy. Am. J. Hum. Genet..

[37] Endo Y., Noguchi S., Hara Y., Hayashi Y.K., Motomura K., Miyatake S., Murakami N., Tanaka S., Yamashita S., Kizu R. (2015). Dominant mutations in ORAI1 cause tubular aggregate myopathy with hypocalcemia via constitutive activation of store-operated Ca^2+^ channels. Hum. Mol. Genet..

[38] Lee J.M., Noguchi S. (2016). Calcium Dyshomeostasis in Tubular Aggregate Myopathy. Int. J. Mol. Sci..

[39] Nesin V., Wiley G., Kousi M., Ong E.C., Lehmann T., Nicholl D.J., Suri M., Shahrizaila N., Katsanis N., Gaffney P.M. (2014). Activating mutations in STIM1 and ORAI1 cause overlapping syndromes of tubular myopathy and congenital miosis. Proc. Natl. Acad. Sci. USA.

[40] Okuma H., Saito F., Mitsui J., Hara Y., Hatanaka Y., Ikeda M., Shimizu T., Matsumura K., Shimizu J., Tsuji S. (2016). Tubular aggregate myopathy caused by a novel mutation in the cytoplasmic domain of STIM1. Neurol. Genet..

[41] Walter M.C., Rossius M., Zitzelsberger M., Vorgerd M., Muller-Felber W., Ertl-Wagner B., Zhang Y., Brinkmeier H., Senderek J., Schoser B. (2015). 50 years to diagnosis: Autosomal dominant tubular aggregate myopathy caused by a novel STIM1 mutation. Neuromuscul. Disord..

[42] Barone V., Del Re V., Gamberucci A., Polverino V., Galli L., Rossi D., Costanzi E., Toniolo L., Berti G., Malandrini A. (2017). Identification and characterization of three novel mutations in the CASQ1 gene in four patients with tubular aggregate myopathy. Hum. Mutat..

[43] Vattemi G.N.A., Rossi D., Galli L., Catallo M.R., Pancheri E., Marchetto G., Cisterna B., Malatesta M., Pierantozzi E., Tonin P. (2022). Ryanodine receptor 1 (RYR1) mutations in two patients with tubular aggregate myopathy. Eur. J. Neurosci..

[44] Engel W.K., Bishop D.W., Cunningham G.G. (1970). Tubular aggregates in type II muscle fibers: Ultrastructural and histochemical correlation. J. Ultrastruct. Res..

[45] Boncompagni S., Protasi F., Franzini-Armstrong C. (2012). Sequential stages in the age-dependent gradual formation and accumulation of tubular aggregates in fast twitch muscle fibers: SERCA and calsequestrin involvement. Age.

[46] Chevessier F., Bauche-Godard S., Leroy J.P., Koenig J., Paturneau-Jouas M., Eymard B., Hantai D., Verdiere-Sahuque M. (2005). The origin of tubular aggregates in human myopathies. J. Pathol..

[47] Salviati G., Pierobon-Bormioli S., Betto R., Damiani E., Angelini C., Ringel S.P., Salvatori S., Margreth A. (1985). Tubular aggregates: Sarcoplasmic reticulum origin, calcium storage ability, and functional implications. Muscle Nerve.

[48] Butler-Browne G.S., Whalen R.G. (1984). Myosin isozyme transitions occurring during the postnatal development of the rat soleus muscle. Dev. Biol..

[49] Paolini C., Quarta M., Wei-LaPierre L., Michelucci A., Nori A., Reggiani C., Dirksen R.T., Protasi F. (2015). Oxidative stress, mitochondrial damage, and cores in muscle from calsequestrin-1 knockout mice. Skelet. Muscle.

[50] Hakim C.H., Li D., Duan D. (2011). Monitoring murine skeletal muscle function for muscle gene therapy. Methods Mol. Biol..

[51] Pietrangelo L., D’Incecco A., Ainbinder A., Michelucci A., Kern H., Dirksen R.T., Boncompagni S., Protasi F. (2015). Age-dependent uncoupling of mitochondria from Ca^2+^ release units in skeletal muscle. Oncotarget.

[52] Pietrangelo L., Michelucci A., Ambrogini P., Sartini S., Guarnier F.A., Fusella A., Zamparo I., Mammucari C., Protasi F., Boncompagni S. (2019). Muscle activity prevents the uncoupling of mitochondria from Ca^2+^ Release Units induced by ageing and disuse. Arch. Biochem. Biophys..

[53] Balaban R.S., Nemoto S., Finkel T. (2005). Mitochondria, oxidants, and aging. Cell.

[54] Chandwaney R., Leichtweis S., Leeuwenburgh C., Ji L.L. (1998). Oxidative stress and mitochondrial function in skeletal muscle: Effects of aging and exercise training. Age.

[55] Leick L., Plomgaard P., Gronlokke L., Al-Abaiji F., Wojtaszewski J.F., Pilegaard H. (2010). Endurance exercise induces mRNA expression of oxidative enzymes in human skeletal muscle late in recovery. Scand. J. Med. Sci. Sports.

[56] Peterson C.M., Johannsen D.L., Ravussin E. (2012). Skeletal muscle mitochondria and aging: A review. J. Aging Res..

[57] Protasi F., Pietrangelo L., Boncompagni S. (2021). Improper Remodeling of Organelles Deputed to Ca^2+^ Handling and Aerobic ATP Production Underlies Muscle Dysfunction in Ageing. Int. J. Mol. Sci..

[58] Shigenaga M.K., Hagen T.M., Ames B.N. (1994). Oxidative damage and mitochondrial decay in aging. Proc. Natl. Acad. Sci. USA.

[59] Trounce I., Byrne E., Marzuki S. (1989). Decline in skeletal muscle mitochondrial respiratory chain function: Possible factor in ageing. Lancet.

[60] Zampieri S., Pietrangelo L., Loefler S., Fruhmann H., Vogelauer M., Burggraf S., Pond A., Grim-Stieger M., Cvecka J., Sedliak M. (2015). Lifelong physical exercise delays age-associated skeletal muscle decline. J. Gerontol. A Biol. Sci. Med. Sci..

[61] Andersson D.C., Betzenhauser M.J., Reiken S., Meli A.C., Umanskaya A., Xie W., Shiomi T., Zalk R., Lacampagne A., Marks A.R. (2011). Ryanodine receptor oxidation causes intracellular calcium leak and muscle weakness in aging. Cell Metab..

[62] Kurebayashi N., Ogawa Y. (2001). Depletion of Ca^2+^ in the sarcoplasmic reticulum stimulates Ca^2+^ entry into mouse skeletal muscle fibres. J. Physiol..

[63] Launikonis B.S., Rios E. (2007). Store-operated Ca^2+^ entry during intracellular Ca^2+^ release in mammalian skeletal muscle. J. Physiol..

[64] Zhang H., Bryson V.G., Wang C., Li T., Kerr J.P., Wilson R., Muoio D.M., Bloch R.J., Ward C., Rosenberg P.B. (2021). Desmin interacts with STIM1 and coordinates Ca^2+^ signaling in skeletal muscle. JCI Insight.

[65] Antelo-Iglesias L., Picallos-Rabina P., Estevez-Souto V., Da Silva-Alvarez S., Collado M. (2021). The role of cellular senescence in tissue repair and regeneration. Mech. Ageing Dev..

[66] Lopez-Otin C., Blasco M.A., Partridge L., Serrano M., Kroemer G. (2013). Hallmarks of aging: An expanding universe. Cell.

[67] Vijg J., Campisi J. (2008). Puzzles, promises and a cure for ageing. Nature.

[68] Luff A.R. (1998). Age-associated changes in the innervation of muscle fibers and changes in the mechanical properties of motor units. Ann. N. Y. Acad. Sci..

[69] Mosole S., Carraro U., Kern H., Loefler S., Fruhmann H., Vogelauer M., Burggraf S., Mayr W., Krenn M., Paternostro-Sluga T. (2014). Long-term high-level exercise promotes muscle reinnervation with age. J. Neuropathol. Exp. Neurol..

