# Supplementary material for: Constitutive, Muscle-Specific Orai1 Knockout Results in the Incomplete Assembly of Ca2+ Entry Units and a Reduction in the Age-Dependent Formation of Tubular Aggregates
Source: Biomedicines. 2024 Jul 24;12(8):1651. doi: 10.3390/biomedicines12081651 (PMC11351919; doi:10.3390/biomedicines12081651)
Supplement: Supplementary file 1 [file biomedicines-12-01651-s001.zip › biomedicines-3054339-supplementary.pdf]

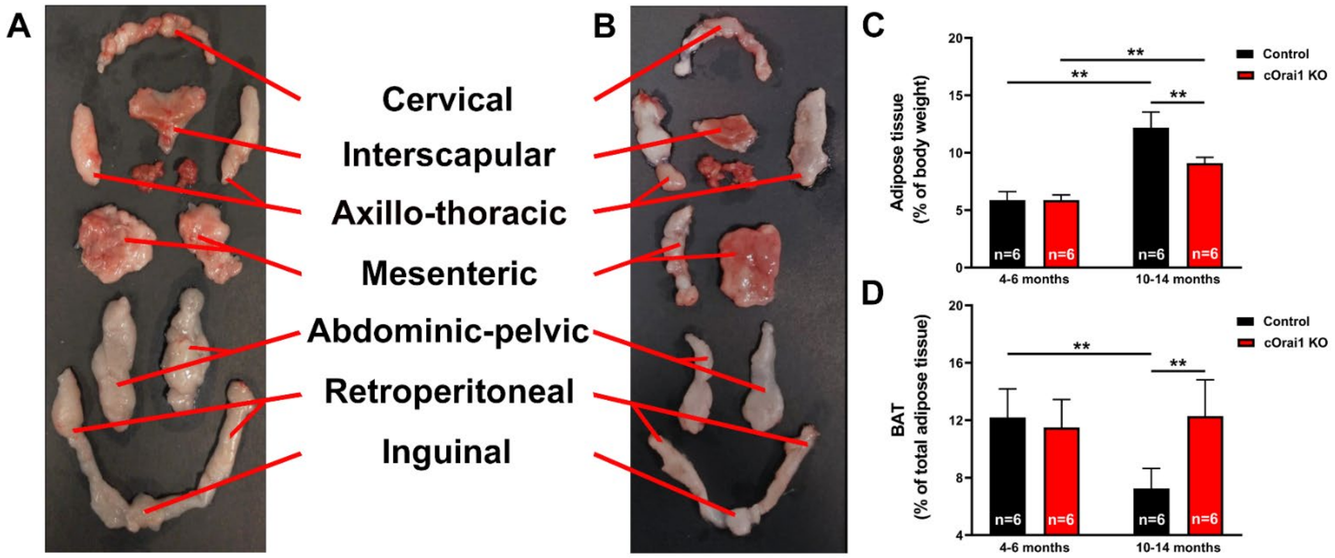

Supplementary Figure S1. Adipose tissue in cOrai1 KO mice. (**A**,**B**) Representative images of adipose tissue dissected from 4 months old control (**A**) and cOrai1 KO (**B**) mice. (**C**) Total body fat expressed as a percentage of body weight. (**D**) Average amount of brown adipose tissue (BAT), expressed as percentage of total adipose tissue. Data are shown as mean ± SEM. ** $p < 0.01$, as evaluated by one-way ANOVA followed by post-hoc Tukey's multiple comparisons test. n = number of mice.
